# Ultrathin Ni_1−_*_x_*Co*_x_*S_2_ nanoflakes as high energy density electrode materials for asymmetric supercapacitors

**DOI:** 10.3762/bjnano.10.213

**Published:** 2019-11-11

**Authors:** Xiaoxiang Wang, Teng Wang, Rusen Zhou, Lijuan Fan, Shengli Zhang, Feng Yu, Tuquabo Tesfamichael, Liwei Su, Hongxia Wang

**Affiliations:** 1 School of Chemistry, Physics and Mechanical Engineering, Science and Engineering Faculty, Queensland University of Technology, Brisbane, QLD 4001, Australia; 2 College of Chemical Engineering, Zhejiang University of Technology, Hangzhou, China

**Keywords:** electrode materials, high energy density, in situ phase transformation, NiCo sulfide, supercapacitors, ultrathin nanoflakes

## Abstract

Transition metal compounds such as nickel cobalt sulfides (Ni–Co–S) are promising electrode materials for energy storage devices such as supercapacitors owing to their high electrochemical performance and good electrical conductivity. Developing ultrathin nanostructured materials is critical to achieving high electrochemical performance, because they possess rich active sites for electrochemical reactions, shortening the transport path of ions in the electrolyte during the charge/discharge processes. This paper describes the synthesis of ultrathin (around 10 nm) flower-like Ni_1−_*_x_*Co*_x_*S_2_ nanoflakes by using templated NiCo oxides. The as-prepared Ni_1−_*_x_*Co*_x_*S_2_ material retained the morphology of the initial NiCo oxide material and exhibited a much improved electrochemical performance. The Ni_1−_*_x_*Co*_x_*S_2_ electrode material exhibited a maximum specific capacity of 1066.8 F·g^−1^ (533.4 C·g^−1^) at 0.5 A·g^−1^ and a capacity retention of 63.4% at 20 A·g^−1^ in an asymmetric supercapacitor (ASC). The ASC showed a superior energy density of 100.5 Wh·kg^−1^ (at a power density of 1.5 kW·kg^−1^), an ultrahigh power density of 30 kW·kg^−1^ (at an energy density of 67.5 Wh·kg^−1^) and excellent cycling stability. This approach can be a low-cost way to mass-produce high-performance electrode materials for supercapacitors.

## Introduction

Supercapacitors (SCs) or electrochemical capacitors (ECs) are regarded as important energy storage devices that provide instantaneous power output to run cranes, subways or trains. They exhibit high power density, long cycling lifetime and fast charge/discharge rates [1–2]. Currently, carbon-based electrical double-layer capacitors (EDLCs) are commercially available, but they exhibit only a low energy density because of physical charge storage processes due to the adsorption/desorption of ions at the electrode–electrolyte interfaces [3]. These drawbacks have hindered further application in areas that require high energy densities such as electric vehicles [4]. In recent years, transition metal compounds have been widely studied as high energy density materials for energy storage devices such as supercapacitors. Advantage was taken of the faradaic redox reaction of transition metal ions and of the low material cost [4]. Nickel–cobalt sulfides have attracted attention as electrode materials for supercapacitors because of their excellent conductivity relative to the oxide counterparts, which contributes to a higher specific capacitance [5]. The multiple valency contributions from both nickel and cobalt ions in the bimetallic sulfides can provide relatively affluent redox reactions, resulting in higher specific capacitance and electrical conductivity [6–7]. Moreover, layered ultrathin nanoflakes in the synthesised nanomaterials, derived from metal oxides/dichalcogenides (TMDs), are attractive as energy-storage electrode materials. This is because the ultrathin structures are fully exposed to the active sites, leading to high electrical conductivity [8–10].

In previous reports, Ni–Co chalcogenides have always been combined with carbon-based materials such as graphene, graphene oxide (GO) or carbon nanotubes (CNTs) in order to improve the charge–discharge process stability [11–13]. There are limited reports regarding a comparison of the intrinsic performance between these Ni–Co chalcogenides materials. Even pure Ni–Co chalcogenide nanomaterials have been designed into different structures in order to investigate the influence on electrode performance. Shen et al. synthesised nickel–cobalt sulfide ball-in-ball hollow spheres through a three-step solvothermal method. The material yielded a capacitance of 1030 F·g^−1^ at 1 A·g^−1^ [14]. Wan et al. reported on NiCo_2_S_4_ nanotubes synthesised via sacrificial template method, which reached a capacitance of 930 F·g^−1^ at 1 A·g^−1^ [15]. Chen et al. reported on one-step electrodeposited nickel–cobalt sulfide nanosheet arrays that reached 1420 F·g^−1^ at 5 A·g^−1^ [16]. Similarly, NiCo_2_O_4_ nanorods with a capacitance of 905 F·g^−1^ at 1 A·g^−1^ were synthesized by Zhang and Lou [17]. Ni–Co mixed oxide nanoprisms with a capacitance of 1000 F·g^−1^ at 10 A·g^−1^ were also reported by Yu and co-workers [18]. Differently designed nanostructures will impact the electrochemical performance of nickel–cobalt oxide/sulfide materials, which makes it difficult to compare the electrochemical performance among the different nanostructures of transition metal chalcogenides.

Herein, we have fabricated ultrathin Ni_1−_*_x_*Co*_x_*S_2_ nanoflakes by sulfurising a NiCo oxide precursor. We have found that the as-prepared Ni_1−_*_x_*Co*_x_*S_2_ nanocomposites well preserved the hierarchical flower-like nanostructures of metal oxides. The material exhibited much higher specific capacitance and rate capability than the NiCo oxide counterparts. The results show that ternary nickel–cobalt sulfides indeed possess better intrinsic electrochemical properties than the corresponding nickel–cobalt oxides with the same material morphology. The assembled ASC also exhibits a superior energy density and high rate capability in a 2 M KOH aqueous electrolyte, making it a promising electrode for SCs.

## Experimental

### Materials

Nickel nitrate hexahydrate (Ni(NO_3_)_2_·6H_2_O), cobalt nitrate hexahydrate (Co(NO_3_)_2_·6H_2_O), 2-methylimidazole (2MI), sulfur powder, absolute methanol and all other reagents were analytical grade and used as received without further purification unless otherwise stated. Deionised water was provided by a Milli-Q water system. YP-50F activated carbon was bought from Kuraray without any purification. All other chemicals were purchased from Sigma-Aldrich.

#### Synthesis of Ni_1.7_Co_1.3_O_4_ powder precursor

The Ni_1.7_Co_1.3_O_4_ precursor was prepared by a facile two-step method. In a solvothermal procedure, similarly to [19], 10 mmol·L^−1^ nickel nitrate hexahydrate, 10 mmol·L^−1^ cobalt nitrate hexahydrate and 40 mmol·L^−1^ 2MI were first dissolved in absolute methanol, and then mixed to make a total volume of 60 mL solution. After stirring at 400 rpm for 10 to 15 min, the well-mixed solution was transferred into a 120 mL Teflon-lined stainless steel autoclave reactor. The reaction was carried out at 120 °C for 14 h. After cooling to room temperature, the as-prepared precipitates were rinsed with absolute methanol at least four times till the pink–purple colour of the solution turned into yellow. The precipitates were fully dried in air at 75 °C for 12 h before being ground to powder. Ni_1.7_Co_1.3_O_4_ powder was obtained via heating the powder in air at 350 °C for 2 h.

#### Fabrication of Ni_1−_*_x_*Co*_x_*S_2_ electrode

Ni_1−_*_x_*Co*_x_*S_2_ was synthesised in a rapid thermal processing (RTP) tube furnace. A graphite box containing about 20 mg as-prepared Ni_1.7_Co_1.3_O_4_ and sufficient sulfur powder (200 mg) was placed in the centre of the RTP tube furnace. The furnace was then evacuated to about 50 mTorr and flushed with argon gas to 13 Torr for three times. After this, the samples were heated in the temperature range of 200–300 °C at a heating rate of 5 °C·min^−1^ for 2 h. The Ni_1−_*_x_*Co*_x_*S_2_ powder was collected after the furnace cooled down to room temperature. For the electrode fabrication, the resultant Ni_1−_*_x_*Co*_x_*S_2_ powder was mixed uniformly with acetylene black and polyvinylidene fluoride (PVDF) with a mass ratio of 8:1:1. The powder mixture was then dispersed in *N*-methyl-2-pyrrolidinone (NMP) and mixed well before being pasted on a pre-cleaned Ni foam substrate (NF). The Ni_1−_*_x_*Co*_x_*S_2_ electrode (Ni_1−_*_x_*Co*_x_*S_2_/NF) was dried in a vacuum oven for 12 h at 80 °C and pressed under 3000 kg·cm^−2^ pressure for 30 s before the electrochemical test. The load mass of the as-prepared materials was in the range of 0.8–1.2 mg·cm^−2^. Notably, the influence of the substrate on the electrochemical performance can be neglected because of its small area of CV curves under the same scan rate (Figure S1, Supporting Information File 1).

#### Material characterization

Field-emission scanning electron microscopy (FESEM, JSM-7001F, JEOL) with energy-dispersive spectrometry (EDS) using an accelerating voltage of 15 kV, and transmission electron microscopy (TEM, JEOL 2100) were used to study the morphology, structure and elemental distributions of the samples. Elemental composition of the as-prepared materials and valence states of each element were analysed using X-ray photoelectron spectroscopy (XPS, Kratos AXIS Supra photoelectron spectrometer, Al Kα excitation (1486.6 eV)). Crystalline structure and composition of the samples were characterized by powder X-ray diffraction analysis (XRD, PANaytical MPD) using a Cu Kα (8047.8 eV) radiation source. Specific surface area and pore size of the as-synthesized material were determined by BET measurements (Micromeritics 3 Flex).

### Electrochemical measurements

#### Electrochemical properties of the electrode material

The electrochemical performance of all samples was tested using a three-electrode system, consisting of a working electrode, a counter electrode of platinum foil and a Hg/HgO reference electrode in 2 mol·L^−1^ potassium hydroxide aqueous electrolyte. The electrochemical characteristics of the synthesised material were measured using an electrochemical workstation (VSP, BioLogic Science Instruments) at room temperature of 24 °C. Electrochemical impedance spectroscopy (EIS) of the electrode material was carried out in the frequency range from 100 kHz to 0.01 Hz at AC voltage under open-circuit conditions. The cycling stability was tested by using a LAND system (Wuhan LAND electronics).

The specific capacitance (*C*_m_, F·g^−1^) and corresponding specific capacity (*C*_a_, mAh·g^−1^) of the as-prepared electrode material were calculated from the galvanostatic charge/discharge (GCD) curves by Equation 1 [20–21] and Equation 2 [22]:

[1] $$C_{\text{m}}=\frac{I\Delta t}{m\Delta V}$$

[2] $$C_{\text{a}}=\frac{C_{\text{m}}\Delta V}{3.6}$$

Where *I* (A) is the discharge current, *t* (s) is discharge time, *m* (g) is the loading mass of the active material on the electrode, Δ*V* is the potential window and Δ*V*/Δ*t* is the slope of the discharge curve. In order to compare with other materials, the specific capacity was determined as *C* (C·g^−1^) = *I*Δ*t*/*m*.

#### Assembly of asymmetric supercapacitors

To acquire the energy density and power density, Ni_1−_*_x_*Co*_x_*S_2_/NF as a positive electrode and activated carbon (AC)/NF as the negative electrode were assembled to an ASC. The mass ratio between positive and negative electrode was calculated according to the charge balance equation (Q^+^ = Q^−^). In order to satisfy the Q^+^ = Q^−^, the mass ratio (active materials) of the two electrodes was determined by Equation 3 [23]:

[3] $$\frac{m_{+}}{m_{-}}=\frac{C_{-}\times \Delta V_{-}}{C_{+}\times \Delta V_{+}}$$

Where *C*_+_ and *C*_−_ (mAh·g^−1^) are the mass-specific capacity of the Ni_1−_*_x_*Co*_x_*S_2_ and AC, respectively. The mass ratio was calculated of the Ni_1−_*_x_*Co*_x_*S_2_ to AC as 1:7.

Energy density *E* (Wh·kg^−1^) and power density *P* (W·kg^−1^) of ASCs were calculated according to Equation 4 and Equation 5 [24]:

[4] $$E=\frac{1}{2}C_{\text{m}}\times \Delta V^{2}\times \frac{1}{3.6}$$

[5] $$P=\frac{E}{\Delta t}\times 3600$$

Δ*V* (V) and Δ*t* (s) represent the potential range and the discharging period of the ASC, respectively. The capacitance *C*_m_ (F·g^−1^) is specific capacitance based on the mass loading of active materials in both electrodes.

## Results and Discussion

**Figure 1:**
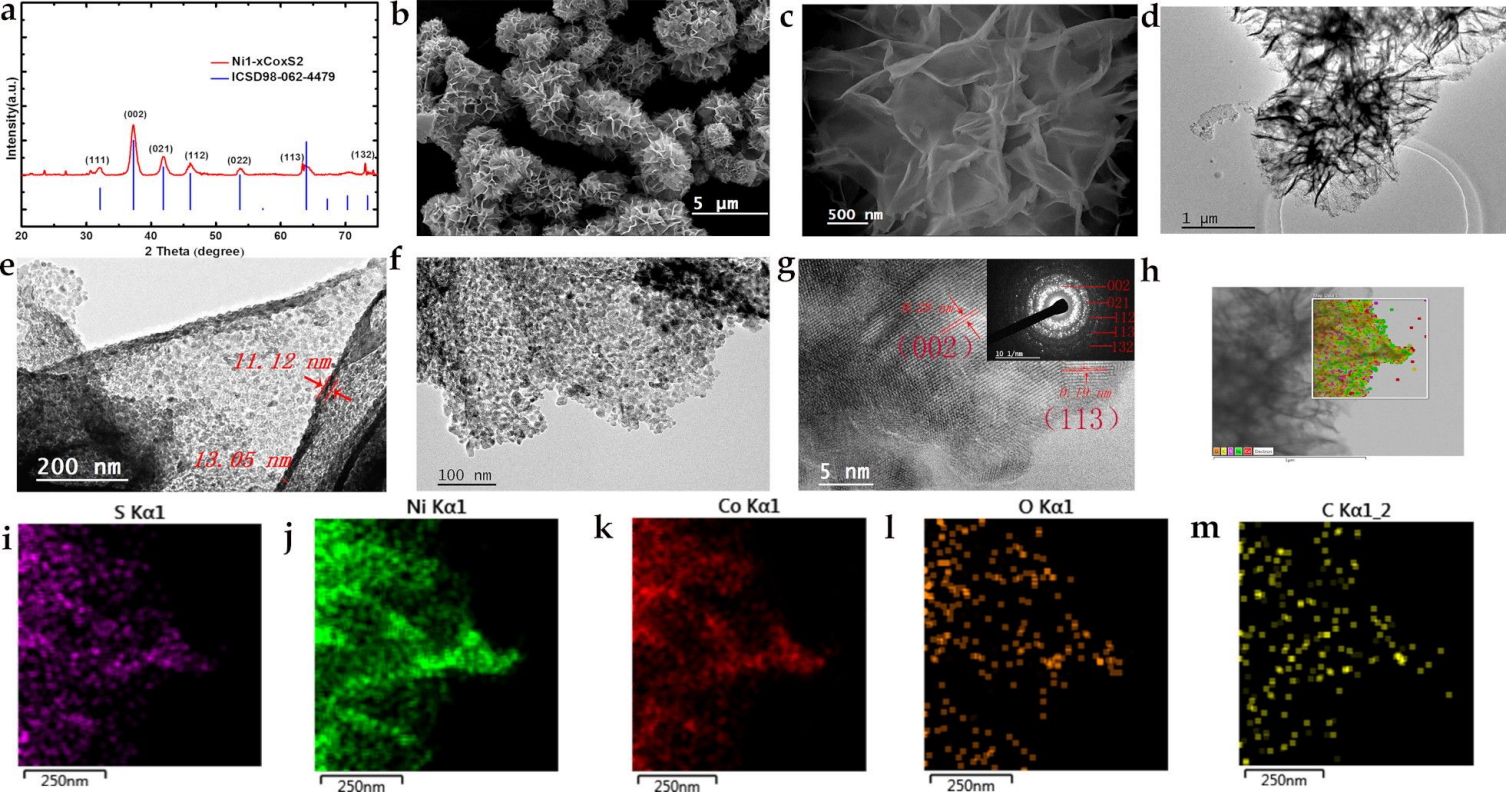
(a) X-ray diffraction patterns of Ni_1−_*_x_*Co*_x_*S_2_; (b) FESEM images and (c) enlarged FESEM images of Ni_1−_*_x_*Co*_x_*S_2_ nanoparticles; (d–g) TEM, HRTEM and SAED pattern (inset) of the Ni_1−_*_x_*Co*_x_*S_2_ nanoflakes; (i–m) EDS elements maps of S, Ni, Co, O and C from the image (h).

Composition and crystal structure of Ni_1.7_Co_1.3_O_4_ were characterized by XRD. The XRD pattern of the NiCo oxides (Supporting Information File 1, Figure S2a) show distinctive diffraction peaks at 2θ = 21.87°, 36.32°, 42.69°, 51.95°, 69.89° and 76.83°, which can be assigned to the (111), (220), (311), (400), (511) and (440) planes of a Ni_1.7_Co_1.3_O_4_ standard sample (PDF#40-1191) [25]. Figure 1a shows the XRD patterns of the sulfurized materials from NiCo oxides, which show relatively sharp diffraction peaks at 32.04°, 37.19°, 41.82°, 46.03°, 53.84° and 64.12° and can be assigned to the (111), (002), (021), (112), (022) and (113) planes of Ni_1−_*_x_*Co*_x_*S_2_ (ICSD98-062-4479) [26], respectively. The relatively broad diffraction peaks are attributed to the small crystallite size of the samples [27].

The morphology of the active materials plays a vital role in influencing their electrochemical performance. Porous structures with rich channels for diffusion of electrolyte ions and large surface area accessible for electrolyte ions favour the electrochemical performance of electrode materials. The morphology of the material was first investigated by SEM. From the SEM images in Figure 1, a flower-like nanostructure that is composed of interconnected nanoflakes (Figure 1b) with plenty of linked and wrinkled nanosheets (Figure 1c) can be observed. The TEM images of the Ni_1−_*_x_*Co*_x_*S_2_ nanosheets (Figure 1d–g) show a highly folded and contorted morphology, indicative of the ultrathin nature of these nanoflakes. The thickness of Ni_1−_*_x_*Co*_x_*S_2_ nanoflakes is estimated to around 10–15 nm based on the folded edges (Figure 1e). The Ni_1−_*_x_*Co*_x_*S_2_ flower-like nanoflakes consisting of numerous interconnected nanoparticles (Figure 1d). This is consistent with the FESEM images of Figure 1b and Figure 1c, which also prove that the structure of the precursor Ni_1.7_Co_1.3_O_4_ (Supporting Information File 1, Figure S2b–d) is maintained through this facile calcination method. Similarly, Ni_1.7_Co_1.3_O_4_ inherits the overall morphology of pristine NiCo-LDHs [19], which proves the microstructure was maintained during the calcination process. Figure 1f also shows that Ni_1−_*_x_*Co*_x_*S_2_ is composed of nano-sized ultrathin crystal grown side by side. Lattice fringe spacings of around 0.28 and 0.19 nm, which can be indexed to the (002) and (113) planes of nickel–cobalt sulfide, respectively, were measured by using high-resolution TEM (HRTEM, Figure 1g). Moreover, the polycrystalline nature of the synthesized Ni–Co sulfides was revealed by the selected area electron diffraction (SAED) pattern (Figure 1g, inset), the diffraction pattern can be readily indexed to the (002), (021), (112), (113) and (132) planes of Ni_1−_*_x_*Co*_x_*S_2_. The diffraction pattern is consistent with the above XRD results. The EDS elemental maps of Ni_1−_*_x_*Co*_x_*S_2_ (Figure 1i–m) show the distribution of S, Ni, Co, O and C and indicates the compositional uniformity of the Ni_1−_*_x_*Co*_x_*S_2_ nanoflakes. Consistent SEM-EDS elemental mappings (Supporting Information File 1, Figure S3) show that Ni and Co are distributed over the whole nanoflakes.

The obtained specific surface area and average pore volume of the Ni_1−_*_x_*Co*_x_*S_2_ nanoflakes are 32.2 m^2^·g^−1^ and 0.18 cm^3^·g^−1^, respectively (Supporting Information File 1, Figure S4a). In comparison, a surface area of 120 m^2^·g^−1^ and a pore volume of 0.84 cm^3^·g^−1^ were obtained for the Ni_1.7_Co_1.3_O_4_ material (Figure S4c, Supporting Information File 1). The relatively low surface area of the Ni_1−_*_x_*Co*_x_*S_2_ nanoflakes is ascribed to the filling with anions (SO_3_^2−^, S^−^, OH^−^), the growth of primary nanoparticles with the subsequent reduction of porosity, which impedes adsorption and desorption of nitrogen molecules [28]. N_2_ molecules can only access the exposed exterior surface, whereas the interlayer space can be easily accessed by electrolyte ions during the charge/discharge process [29]. Additionally, Ni_1−_*_x_*Co*_x_*S_2_ possesses a small pore sizes distribution with a mean pore size of about 1.5 nm (Figure S4b, Supporting Information File 1). This result verifies that the pore size of the three-dimensional structure of the Ni_1−_*_x_*Co*_x_*S_2_ nanoflakes is smaller than the pore size of Ni_1.7_Co_1.3_O_4_ (around 4 nm) as shown in Figure S4d (Supporting Information File 1). The pores in the material can shorten the ion diffusion path lengths, overcoming the kinetics barrier of the faradaic redox reaction for most of the energy storage materials. The hierarchical nanostructure can provide abundant surface active sites for electrochemical reactions during the charge/discharge process.

**Figure 2:**
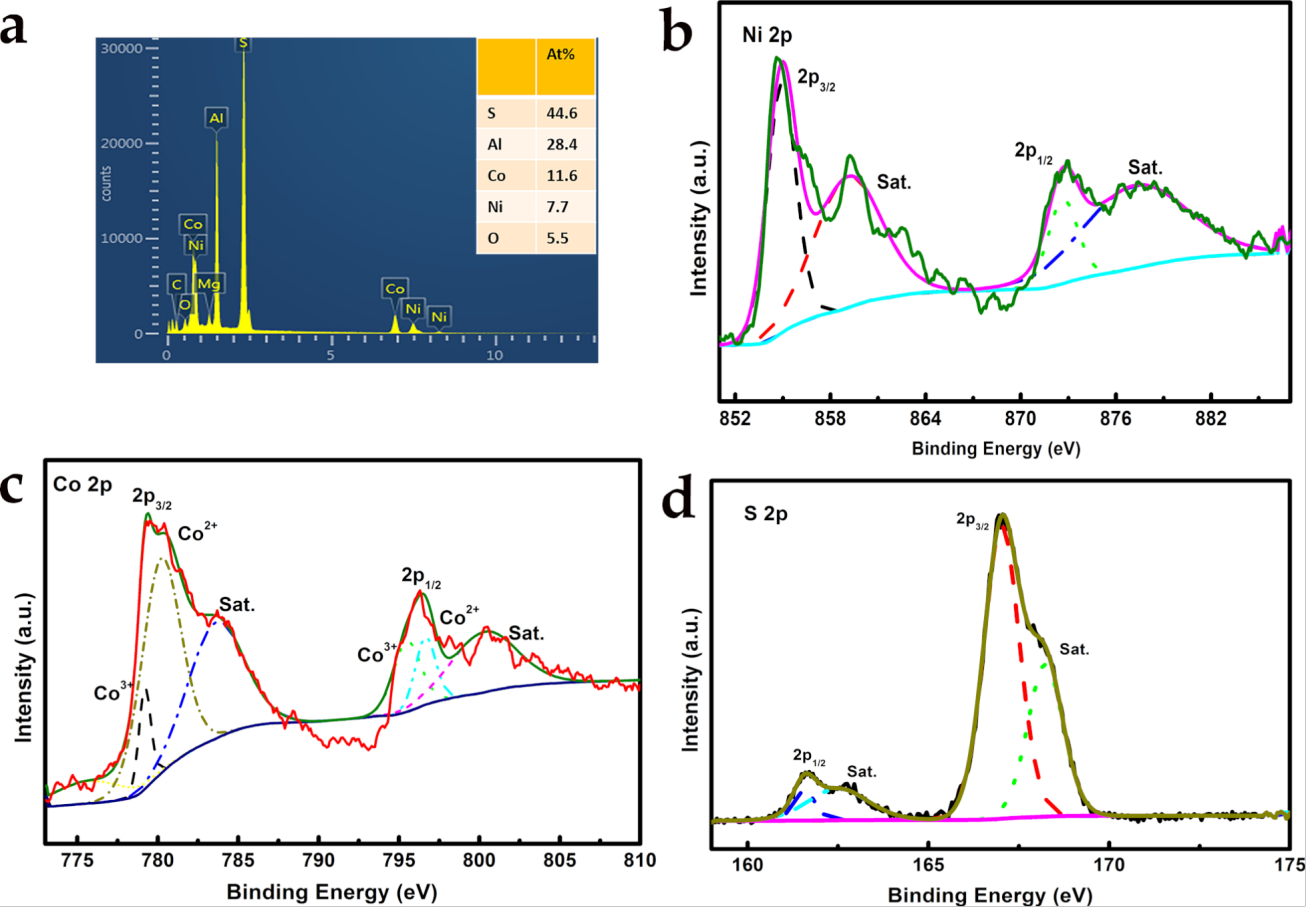
(a) EDS pattern of Ni_1−_*_x_*Co*_x_*S_2_ and high-resolution XPS spectra of (b) Ni 2p, (c) Co 2p, and (d) S 2p for Ni_1−_*_x_*Co*_x_*S_2_.

The elemental composition of Ni_1−_*_x_*Co*_x_*S_2_, is shown in Figure 2a. Elements including Co, Ni, S, and O are detected, confirming the successful incorporation of sulfur in the material after sulfurization with a Ni/Co/S ratio of 1:1.5:5.8, respectively. The high content of Al is because the samples are supported by aluminium SEM sample stages. Chemical composition and valence states of each element in the material are further confirmed by using XPS. The high-resolution XPS spectra confirm the existence of nickel, cobalt, and sulfur. After fitting the XPS with the Gaussian method, the HRXPS spectrum of Ni 2p_1/2_ and Ni 2p_3/2_ (Figure 2b) with binding energies of 855.9 eV and 873.3 eV are observed, respectively, with an energy gap of 17.4 eV. Two satellite (indicated as “Sat.”) peaks are also observed at 859.9 eV and 878.4 eV. Thus, the existence of Ni^2+^ is confirmed in the sulfurised resultants [30–31]. The Co 2p HRXPS spectrum (Figure 2c) shows characteristic peaks of Co 2p, which were both fitted with two main peaks at 781.4 eV and 796.7 eV, corresponding to a spin-energy separation of 15.3 eV. This energy can be attributed to the existence of Co^3+^ in the Ni_1−_*_x_*Co*_x_*S_2_ [32–33]. In the S 2p spectra shown in Figure 2d, the peaks at 166.1 eV indicate the existence of S^2−^ while the peaks at 161.1 eV can be attributed to SO_3_^2−^ anions. The above results indicate the chemical composition of the as-prepared Ni_1−_*_x_*Co*_x_*S_2_ including Ni^2+^, Co^2+^, Co^3+^, and S^2−^, which is in agreement with previous results of nickel–cobalt sulfides [14,23,30–31,34–35]. The C 1s spectrum of the Ni_1−_*_x_*Co*_x_*S_2_ nanoflakes can be fitted to three peaks of C=C, C=O, and O−C=O (Supporting Information File 1, Figure S5) [36]. The C/O functional surface groups can be ascribed to CO_3_^2−^ in the layers of nanoflakes and to surface carbon residues, contributing to the continuous oxidation of Co^2+^ to Co^3+^ [37].

**Figure 3:**
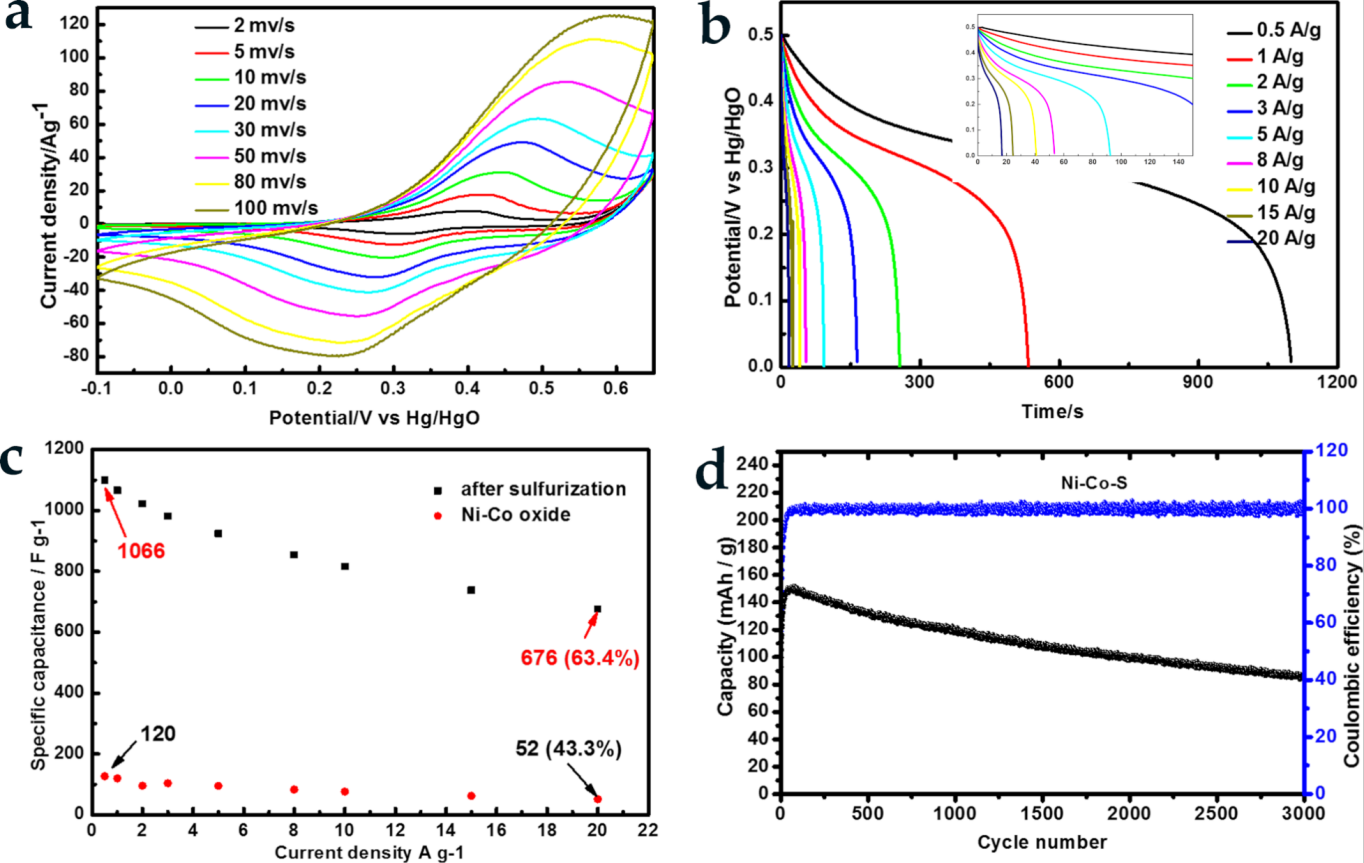
(a) CV curves at sweep rates from 2 to 100 mV·s^−1^ (vs Hg/HgO); (b) GCD curves at different current densities; (c) comparison of specific capacitance retention with precursor Ni*_x_*Co_3−_*_x_*O_4_ and Ni_1−_*_x_*Co*_x_*S_2_; (d) cycle stability of Ni_1−_*_x_*Co*_x_*S_2_.

The electrochemical performance of the as-prepared oxide and sulfide materials was investigated by a three-electrode system at room temperature. A 1 × 1 cm^2^ sample of NiCoS@NF was used as the working electrode while a platinum foil and Hg/HgO electrode were used as a counter and a reference electrode, respectively. The electrolyte was a 2 M aqueous KOH solution. Cyclic voltammetry (CV) curves of Ni_1−_*_x_*Co*_x_*S_2_ at scan rates from 2 to 100 mV·s^−1^ are shown in Figure 3a. As expected, redox peaks observed within the potential window from −0.1 to 0.65 V (vs Hg/HgO), which can be attributed to reversible faradaic redox reactions of Co^2+^/Co^3+^/Co^4+^ and Ni^2+^/Ni^3+^ associated with the following reaction equations (Equation 6–8) [14,31]:

[6] $$\text{CoS}+{\text{OH}}^{-}↔\text{CoSOH}+{\text{e}}^{-}$$

[7] $$\text{CoSOH}+{\text{OH}}^{-}↔\text{CoS}+{\text{H}}_{2}\text{O}+{\text{e}}^{-},$$

[8] $$\text{NiS}+{\text{OH}}^{-}↔\text{NiSOH}+{\text{e}}^{-}.$$

With increased sweep rates, the anodic peaks move in the positive direction and the cathodic peaks move in the negative direction. Moreover, the similar CV curve shapes suggest battery energy storage characteristics and good rate capabilities of the electrode [38]. Figure 3b shows the GCD curves at different current densities, which were used to calculate the specific capacitance based on Equation 1. Because the nickel–cobalt sulfide was specified as a battery-type material, the capacity *C* was also used for comparison with other materials. The specific capacity was defined by *C* (C·g^−1^) = *I*Δ*t*/*m*. As shown in Figure 3c, the specific capacitance of Ni–Co oxides at the current densities of 1, 5, 10, 20 A·g^−1^ are 120 F·g^−1^ (60 C·g^−1^), 95 F·g^−1^ (47.5 C·g^−1^), 76.2 F·g^−1^ (38.1 C·g^−1^), 52 F·g^−1^ (26 C·g^−1^), respectively. The specific capacitance of Ni_1.7_Co_1.3_O_4_ material is calculated from the charge and discharge curve (Supporting Information File 1, Figure S6a). After the sulfurization, the specific capacitance of the electrode material was enhanced to 1066 F·g^−1^ (533 C·g^−1^), 924 F·g^−1^ (462 C·g^−1^), 816 F·g^−1^ (408 C·g^−1^), 676 F·g^−1^ (338 C·g^−1^). This is a nearly ten-fold enhancement in the electrochemical energy storage of Ni_1−_*_x_*Co*_x_*S_2_ compared to the Ni_1.7_Co_1.3_O_4_ counterpart, which can be attributed to the higher conductivity of the sulfide material [39].

Another critical parameter of supercapacitor electrodes is cycling stability. It can be seen from Figure 3d that the capacity increased in the first 50 cycles, which can be due to the wetting process of the electrode in the electrolyte [40]. After a total of 3000 charge/discharge cycles at 10 A·g^−1^, Ni_1−_*_x_*Co*_x_*S_2_ retains 67% of its initial capacity, indicating a good cycling stability. The above results imply that this facile sulfurization method can be used as a universal method for enhancing the electrochemical performance of transition metal oxides.

We further investigated the effect of the sulfurization temperature on the capacitance of the Ni_1−_*_x_*Co*_x_*S_2_ at different temperatures of 200, 250, 300, and 350 °C. As shown in Supporting Information File 1, Figure S6b, the Ni_1−_*_x_*Co*_x_*S_2_ nanoflakes demonstrate the best electrochemical performance after calcination at 300 °C. The Ni–Co sulfides showed a maximum capacitance of 1066.8 F·g^−1^ (533 C·g^−1^) at 0.5 A·g^−1^ and a retention rate of 63.4% when the current density increases to 20 A·g^−1^. The detailed CV and GCD plots of the other three groups are shown in Figure S7 (Supporting Information File 1).

**Figure 4:**
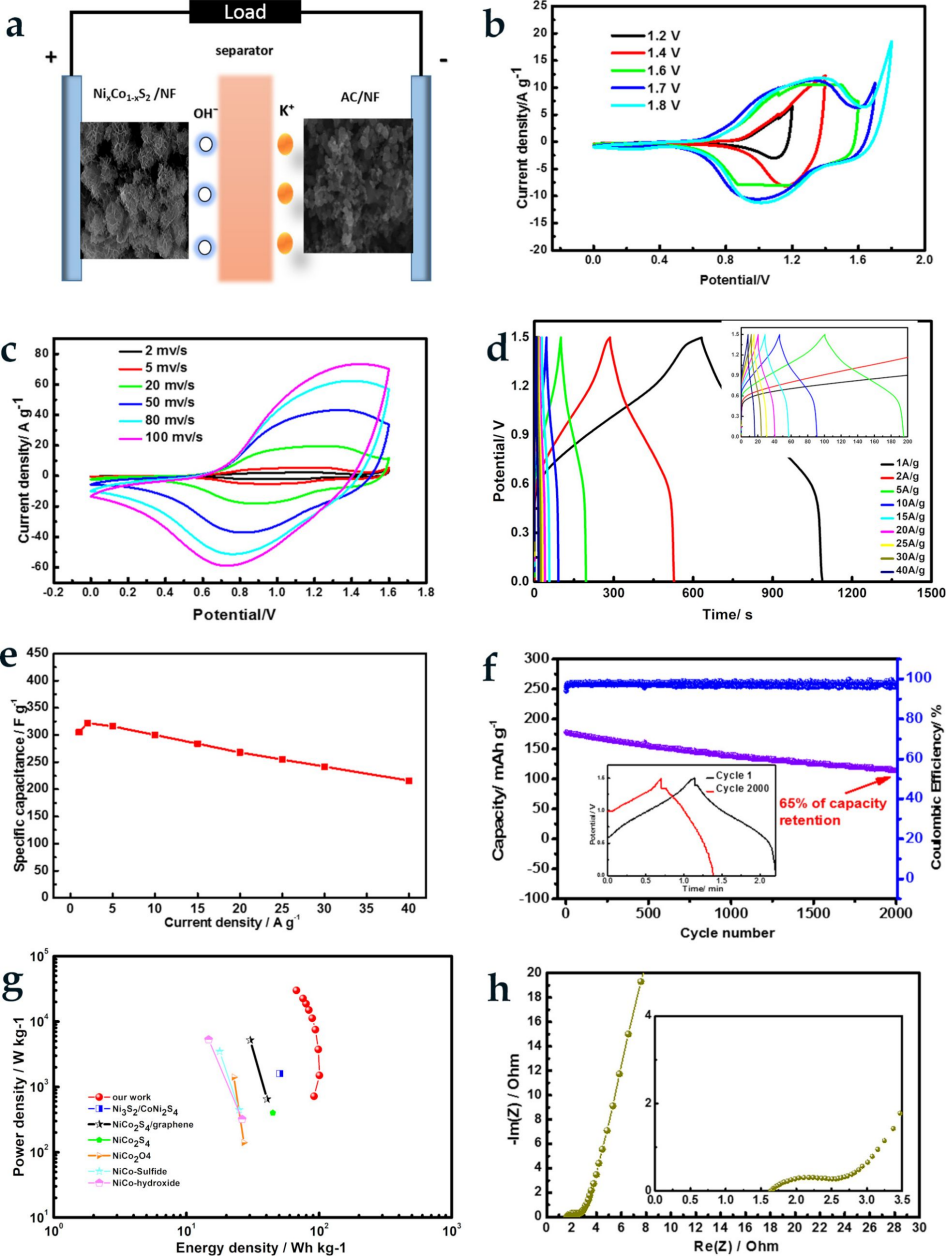
a) Configuration of the ASC; (b) CV curves of the ASC at different potential windows with a scan rate of 10 mV·s^−1^; (c) CV curves of the device at various sweep rates and (d) GCD curves of ASC at different charging/discharge current densities; and (e) the corresponding specific capacitance, (f) cycling stability of the ASC under a current density of 10 A·g^−1^, (g) Ragone plot, and (h) EIS plot of the device.

To evaluate energy density and power density of the Ni_1−_*_x_*Co*_x_*S_2_ material for practical applications, the as-prepared Ni_1−_*_x_*Co*_x_*S_2_/NF as a positive electrode and the commercial YP-50F activated carbon (AC/NF) as a negative electrode (Figure 4a) were assembled to an asymmetric supercapacitor. As shown in Supporting Information File 1, Figure S8a and S8b, the commercial carbon exhibited an approximately rectangular-shaped CV curve and delivered a capacitance of 103 F·g^−1^ at 1 A·g^−1^, making it suitable as a negative electrode. From the CV curves of the ASC device (Figure 4b) scanned at a rate of 10 mV·s^−1^, the usable potential windows were found up to 1.6 V. Polarization occurred when the potential was expanded beyond this value (1.7–1.8 V). The as-fabricated asymmetric supercapacitor showed battery behaviour in the range of 0–1.6 V at different scan rates as shown in Figure 4c. The capacity of the device has both faradaic and EDLC contributions. The small shape change of the CV curves with the increase of scan rate demonstrates the good reversibility of the ASC [28]. The GCD curves (Figure 4d) of the ASC are nonlinear, indicating the faradaic mechanism of the charge storage. Furthermore, a high coulombic efficiency (ca. 100%), electrochemical reversibility and a fast charge transfer process are observed from the nearly symmetric GCD curves. The specific capacitance of the ASC is calculated from the GCD plots. As shown in Figure 4e, the specific capacitance of the ASC reaches 321.6 F·g^−1^ (482.4 C·g^−1^) at a current density of 2 A·g^−1^. Noticeably, a comparatively high value of 216 F·g^−1^ (324 C·g^−1^) was retained even at an extremely heavy current density of 40 A·g^−1^. This further confirms the superior capacitance and high rate performance of the Ni_1−_*_x_*Co*_x_*S_2_/NF//AC/NF asymmetric supercapacitor. The cycling performance of the ASC was evaluated at a large current density of 10 A·g^−1^ shown in Figure 4f. The ASC displays excellent cycling stability with about 65% of the initial specific capacitance even after 2000 cycles. Figure 4g shows the energy density as a function of the power density (Ragone plot) of the as-fabricated ASCs. An extremely high energy density of 100.5 Wh·kg^−1^ at a power density of 1496.5 W kg^−1^ is achieved, with the device maintaining an energy density of 67.5 Wh·kg^−1^ at an extraordinarily high power density of 30 kW·kg^−1^. To visualise the performance of the new ASC device with ultrathin nanoflakes, the results were compared with previously reported NiCo-based ASCs. The new device outperforms Ni_3_S_2_/CoNi_2_S_4_/NF//AC [41], NiCo_2_S_4_/graphene//AC [39], NiCo_2_S_4_/NCF//(OMC = ordered mesoporous carbon)/NCF [31], NiCo_2_O_4_//AC [17], NiCo-sulfide//AC [28] and NiCo-hydroxide//AC [42]. The EIS results of the Ni_1−_*_x_*Co*_x_*S_2_ material is shown in Figure 4h. The ASC shows a small intercept of 1.7 Ω and semicircle with small radius in the high-frequency part which reveals a good ion transport resistance in the as-prepared ASC. The big slope at low frequencies indicates a fast mass transfer rate in the electrolyte [43]. These results reconfirm the superior performance of the Ni_1−_*_x_*Co*_x_*S_2_/NF//AC/NF ASC and demonstrate a great potential in high power supercapacitors.

## Conclusion

We have demonstrated a facile strategy for the fabrication of Ni_1−_*_x_*Co*_x_*S_2_ ultrathin nanoflakes with the thickness of about 10 nm. This method can also maintain the 3D hierarchical porous nanostructure of precursor materials. The as-prepared sulfide material exhibited improved electrochemical performance with a specific capacitance of 1066.8 F·g^−1^ (533.4 C·g^−1^) at 0.5 A·g^−1^ and a 63.4% capacitance retention at 20 A·g^−1^ and excellent cycling stability (67% after 2000 cycles). The corresponding asymmetric supercapacitor, with Ni_1−_*_x_*Co*_x_*S_2_/NF as the positive electrode and AC/NF as the negative electrode, exhibited a high energy density of 100.5 Wh·kg^−1^ (at a power density of 1.5 kW·kg^−1^), a superior power density of 30 kW·kg^−1^ (at an energy density of 67.5 Wh·kg^−1^), and excellent cycling stability and capacity retention. These results can be credited to synergic effects rich and fast redox reactions, high conductivity, as well as highly porous and robust ultrathin nanoflakes structures.

## Supporting Information

File 1 Additional figures.

## References

[1] Choudhary N, Li C, Moore J, Nagaiah N, Zhai L, Jung Y, Thomas J (2017). Adv Mater (Weinheim, Ger).

[2] Yousaf M, Shi H T H, Wang Y, Chen Y, Ma Z, Cao A, Naguib H E, Han R P S (2016). Adv Energy Mater.

[3] Wang L, Xie X, Dinh K N, Yan Q, Ma J (2019). Coord Chem Rev.

[4] Wu M, Ni W, Hu J, Ma J (2019). Nano-Micro Lett.

[5] Liu C, Wu X, Xia H (2018). CrystEngComm.

[6] Ji H, Liu C, Wang T, Chen J, Mao Z, Zhao J, Hou W, Yang G (2015). Small.

[7] Khani H, Wipf D O (2017). ACS Appl Mater Interfaces.

[8] Zhang G, Liu H, Qu J, Li J (2016). Energy Environ Sci.

[9] Yuan C, Li J, Hou L, Zhang X, Shen L, Lou X W D (2012). Adv Funct Mater.

[10] Zhao Y, Chen G, Bian T, Zhou C, Waterhouse G I N, Wu L-Z, Tung C-H, Smith L J, O'Hare D, Zhang T (2015). Adv Mater (Weinheim, Ger).

[11] Ma H, He J, Xiong D-B, Wu J, Li Q, Dravid V, Zhao Y (2016). ACS Appl Mater Interfaces.

[12] Luan Y, Zhang H, Yang F, Yan J, Zhu K, Ye K, Wang G, Cheng K, Cao D (2018). Appl Surf Sci.

[13] Wang M, Yang J, Liu S, Hu C, Li S, Qiu J (2019). ACS Appl Mater Interfaces.

[14] Shen L, Yu L, Wu H B, Yu X-Y, Zhang X, Lou X W (2015). Nat Commun.

[15] Wan H, Jiang J, Yu J, Xu K, Miao L, Zhang L, Chen H, Ruan Y (2013). CrystEngComm.

[16] Chen W, Xia C, Alshareef H N (2014). ACS Nano.

[17] Zhang G, Lou X W D (2013). Sci Rep.

[18] Yu L, Guan B, Xiao W, Lou X W D (2015). Adv Energy Mater.

[19] Wang T, Zhang S, Yan X, Lyu M, Wang L, Bell J, Wang H (2017). ACS Appl Mater Interfaces.

[20] Li G, Li W, Xu K, Zou R, Chen Z, Hu J (2014). J Mater Chem A.

[21] Sun M, Tie J, Cheng G, Lin T, Peng S, Deng F, Ye F, Yu L (2015). J Mater Chem A.

[22] Wang X, Wang T, Su L, Tesfamichael T, Yu F, Shi Z, Wang H (2019). J Alloys Compd.

[23] Xu S, Su C, Wang T, Ma Y, Hu J, Hu J, Hu N, Su Y, Zhang Y, Yang Z (2018). Electrochim Acta.

[24] Song Y, Xu J-L, Liu X-X (2014). J Power Sources.

[25] Petrov K, Will G (1987). J Mater Sci Lett.

[26] Bouchard R J (1968). Mater Res Bull.

[27] Monshi A, Foroughi M R, Monshi M R (2012). World J Nano Sci Eng.

[28] Li Y, Cao L, Qiao L, Zhou M, Yang Y, Xiao P, Zhang Y (2014). J Mater Chem A.

[29] Cai X, Shen X, Ma L, Ji Z, Xu C, Yuan A (2015). Chem Eng J.

[30] Tran V C, Sahoo S, Shim J-J (2018). Mater Lett.

[31] Shen L, Wang J, Xu G, Li H, Dou H, Zhang X (2015). Adv Energy Mater.

[32] Yang J, Yu C, Fan X, Ling Z, Qiu J, Gogotsi Y (2013). J Mater Chem A.

[33] Wang X, Yan C, Sumboja A, Yan J, Lee P S (2014). Adv Energy Mater.

[34] Yu L, Zhang L, Wu H B, Lou X W D (2014). Angew Chem, Int Ed.

[35] Xiao J, Wan L, Yang S, Xiao F, Wang S (2014). Nano Lett.

[36] Yang J, Yu C, Fan X, Zhao C, Qiu J (2015). Adv Funct Mater.

[37] Yang J, Yu C, Fan X, Liang S, Li S, Huang H, Ling Z, Hao C, Qiu J (2016). Energy Environ Sci.

[38] Lu F, Zhou M, Li W, Weng Q, Li C, Xue Y, Jiang X, Zeng X, Bando Y, Golberg D (2016). Nano Energy.

[39] Yu F, Chang Z, Yuan X, Wang F, Zhu Y, Fu L, Chen Y, Wang H, Wu Y, Li W (2018). J Mater Chem A.

[40] Wang Z, Zhu Z, Zhang Q, Zhai M, Gao J, Chen C, Yang B (2019). Ceram Int.

[41] He W, Wang C, Li H, Deng X, Xu X, Zhai T (2017). Adv Energy Mater.

[42] Sun X, Wang G, Sun H, Lu F, Yu M, Lian J (2013). J Power Sources.

[43] Liu X, Yang Q, Mi M, Kong W, Ge Y, Ma J, Hu J (2019). J Alloys Compd.

