# Supplementary material for: Ultrathin Ni1−xCoxS2 nanoflakes as high energy density electrode materials for asymmetric supercapacitors
Source: Beilstein J Nanotechnol. 2019 Nov 11;10:2207–16. doi: 10.3762/bjnano.10.213 (PMC6880822; doi:10.3762/bjnano.10.213)
Supplement: File 1 — Additional figures. [file Beilstein_J_Nanotechnol-10-2207-s001.pdf]

BEILSTEIN JOURNAL OF NANOTECHNOLOGY

Supporting Information

for

Ultrathin $Ni_{1-x}Co_xS_2$ nanoflakes as high energy density electrode materials for asymmetric supercapacitors

Xiaoxiang Wang, Teng Wang, Rusen Zhou, Lijuan Fan, Shengli Zhang, Feng Yu, Tuquabo Tesfamichael, Liwei Su and Hongxia Wang

Beilstein J. Nanotechnol. **2019,** *10,* 2207–2216. doi:10.3762/bjnano.10.213

Additional figures

License and Terms: This is a supporting information file under the terms of the Creative Commons Attribution License (http://creativecommons.org/licenses/by/4.0). Please note that the reuse, redistribution and reproduction in particular requires that the authors and source are credited.
The license is subject to the *Beilstein Journal of Nanotechnology* terms and conditions: (https://www.beilstein-journals.org/bjnano)

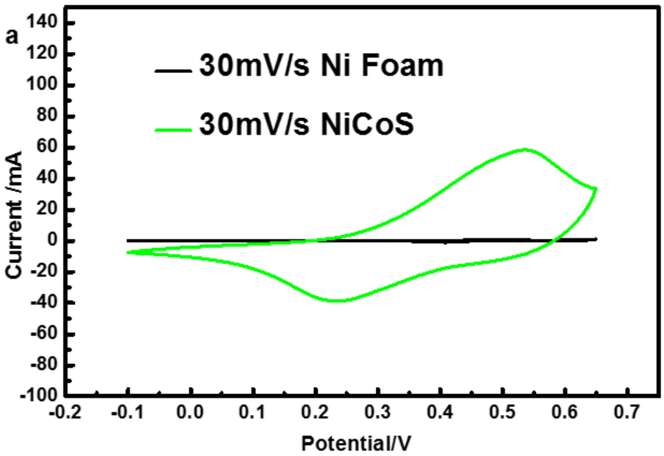

Figure S1: CV (vs Hg/HgO) comparison of bare Ni foam and $Ni_{1-x}Co_xS_2$.

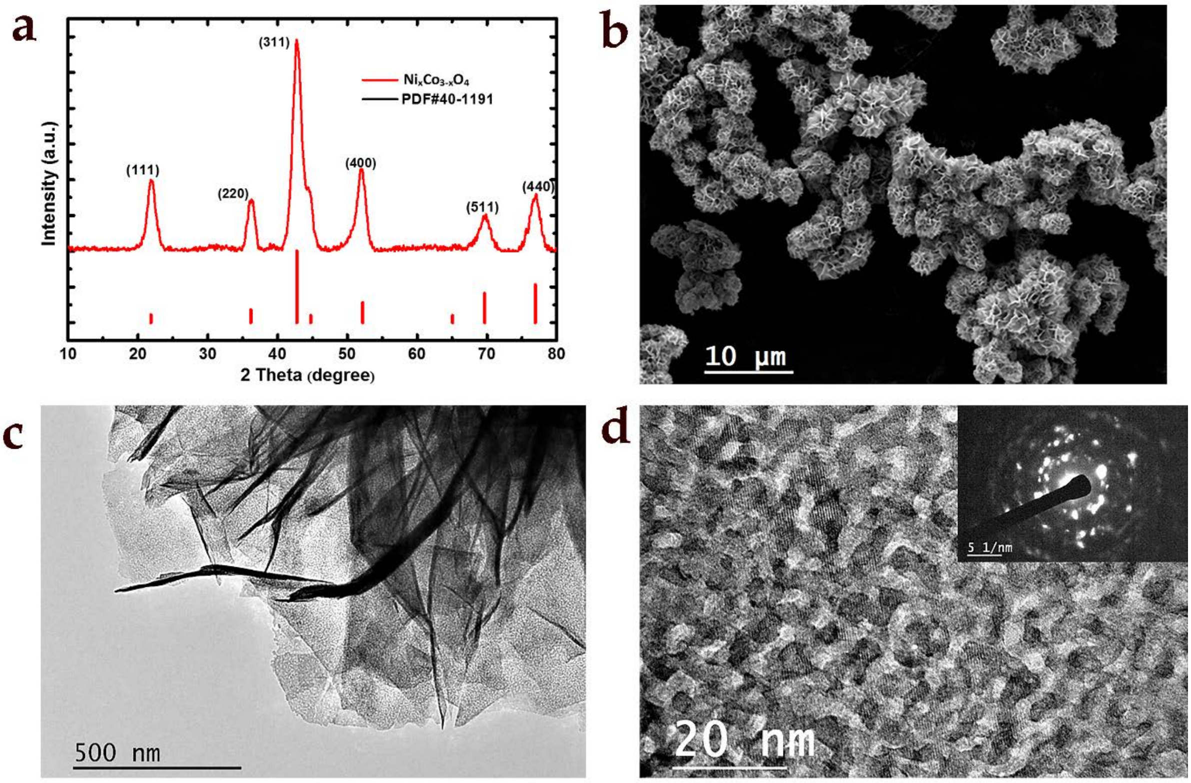

Figure S2: (a) XRD pattern of $Ni_{1.7}Co_{1.3}O_4$ materials; (b) FESEM images of $Ni_{1.7}Co_{1.3}O_4$; (c and d) Low magnification and enlarged TEM images of precursor $Ni_{1.7}Co_{1.3}O_4$, the inset shows the SAED pattern of the materials.

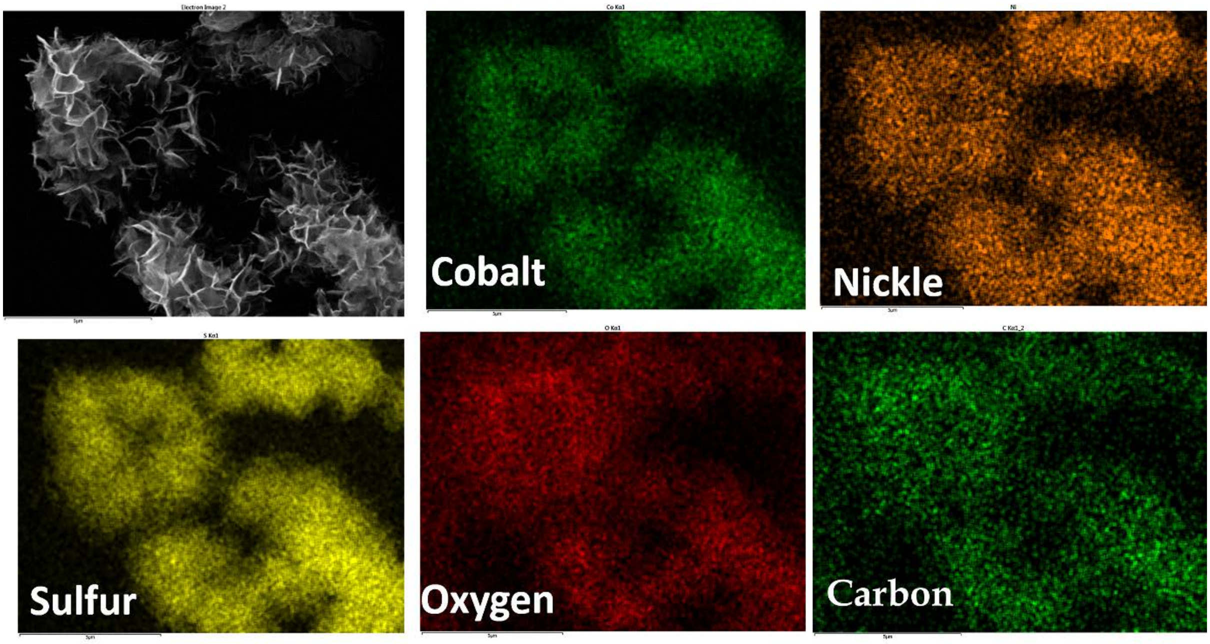

Figure S3: FESEM-EDS mapping of elements in $Ni_{1-x}Co_xS_2$ nano particles.

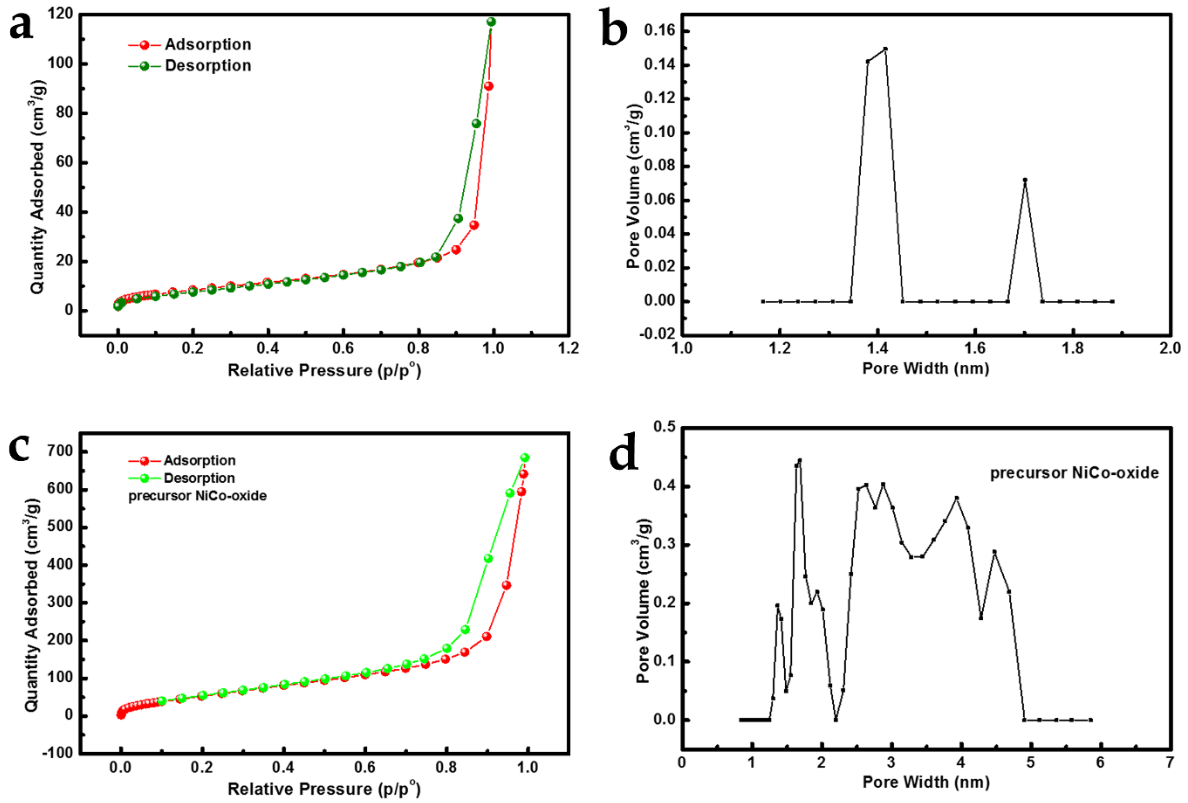

Figure S4: a) N2 adsorption/desorption isotherms and b) pore size distribution plot of $Ni_{1-x}Co_KS_2$ materials; c) N2 adsorption/desorption isotherms and d) pore size distribution plot of $Ni_{1.7}Co_{1.3}O_4$ materials.

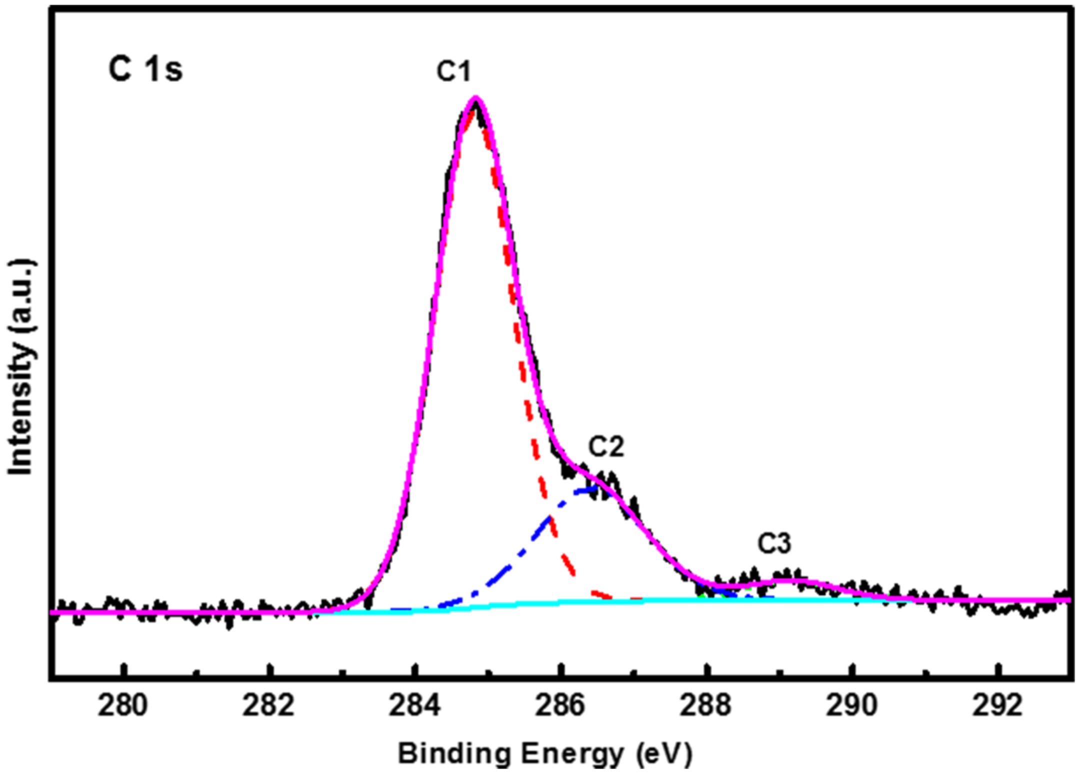

Figure S5: High-resolution XPS spectra C 1s of the $Ni_{1-x}Co_xS_2$ nanoflakes.

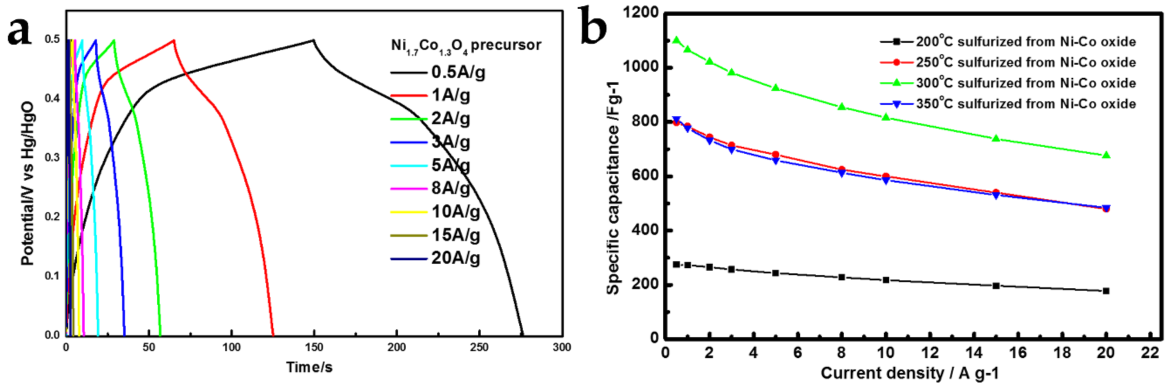

Figure S6: (a) GCD curves of $Ni_{1.7}Co_{1.3}O_4$ materials; (b) Specific capacitance comparison of different sulfurization temperature.

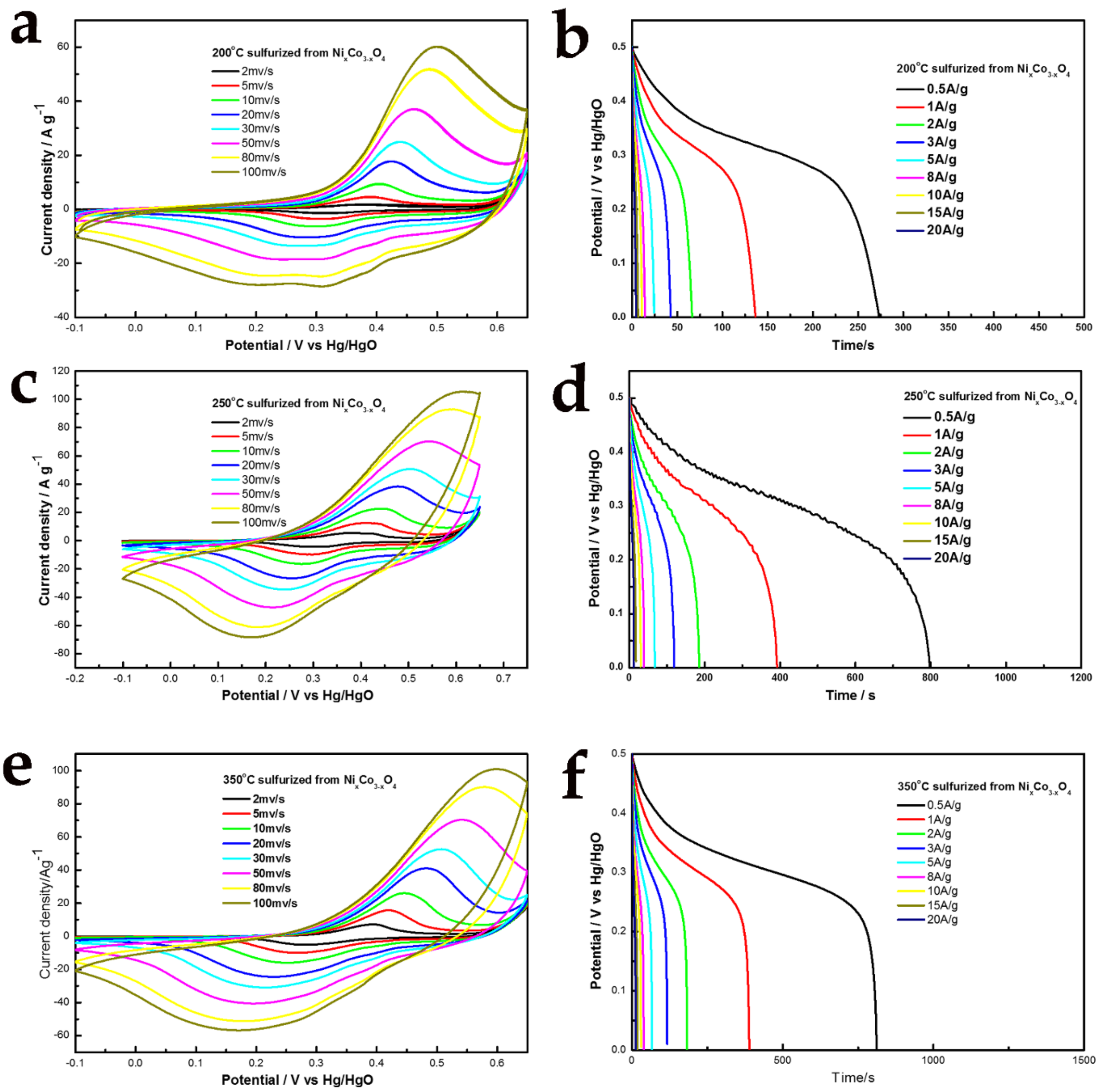

Figure S7: CV and GCD plots (vs Hg / HgO) of $Ni_{1-x}Co_xS_2$ sulfurized under different temperature of 200 °C (a,b); 250 °C (c,d) and 350 °C (e,f).

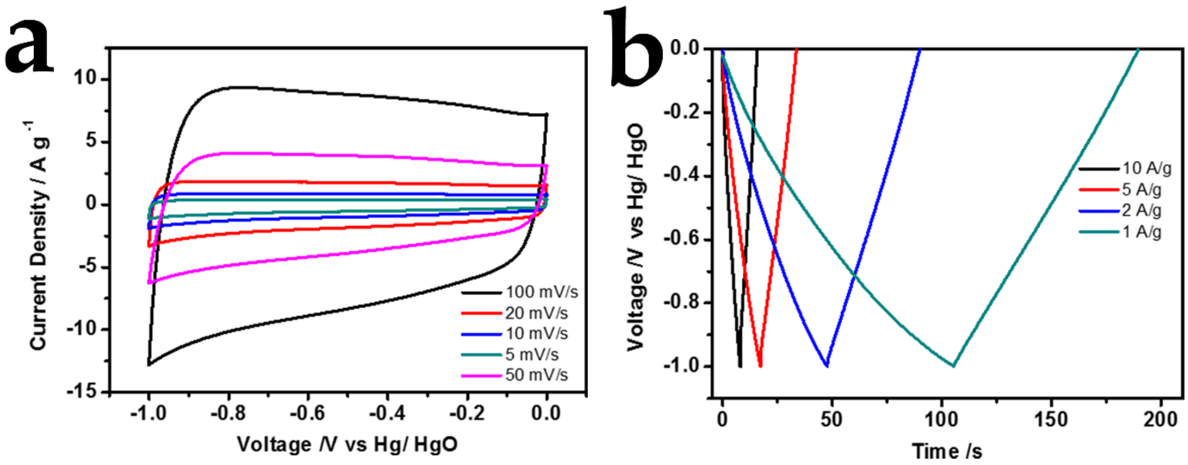

Figure S8: (a) CV and (b) GCD plots (vs Hg / HgO) of negative material YP-50F commercial carbon.
